# Introduction of *in vitro *transcribed *ENO1 *mRNA into neuroblastoma cells induces cell death

**DOI:** 10.1186/1471-2407-5-161

**Published:** 2005-12-16

**Authors:** Katarina Ejeskär, Cecilia Krona, Helena Carén, Faten Zaibak, Lingli Li, Tommy Martinsson, Panayiotis A Ioannou

**Affiliations:** 1 Dept. Clinical Genetics, University of Gothenburg, Sahlgrenska University Hospital/East, SE-416 85 Gothenburg, Sweden; 2 Murdoch Children's Research Institute, Department of Paediatrics, The University of Melbourne, Royal Children's Hospital, Melbourne, VIC 3052, Australia

## Abstract

**Background:**

Neuroblastoma is a solid tumour of childhood often with an unfavourable outcome. One common genetic feature in aggressive tumours is 1p-deletion.

The α-enolase (*ENO1*) gene is located in chromosome region 1p36.2, within the common region of deletion in neuroblastoma. One alternative translated product of the *ENO1 *gene, known as *MBP-1*, acts as a negative regulator of the *c-myc *oncogene, making the *ENO1 *gene a candidate as a tumour suppressor gene.

**Methods:**

Methods used in this study are transfection of cDNA-vectors and *in vitro *transcribed mRNA, cell growth assay, TUNEL-assay, real-time RT-PCR (TaqMan) for expression studies, genomic sequencing and DHPLC for mutation detection.

**Results:**

Here we demonstrate that transfection of *ENO1 *cDNA into 1p-deleted neuroblastoma cell lines causes' reduced number of viable cells over time compared to a negative control and that it induces apoptosis. Interestingly, a similar but much stronger dose-dependent reduction of cell growth was observed by transfection of *in vitro *transcribed *ENO1 *mRNA into neuroblastoma cells. These effects could also be shown in non-neuroblastoma cells (293-cells), indicating *ENO1 *to have general tumour suppressor activity.

Expression of *ENO1 *is detectable in primary neuroblastomas of all different stages and no difference in the level of expression can be detected between 1p-deleted and 1p-intact tumour samples. Although small numbers (11 primary neuroblastomas), there is some evidence that Stage 4 tumours has a lower level of *ENO1*-mRNA than Stage 2 tumours (p = 0.01). However, mutation screening of 44 primary neuroblastomas of all different stages, failed to detect any mutations.

**Conclusion:**

Our studies indicate that *ENO1 *has tumour suppressor activity and that high level of *ENO1 *expression has growth inhibitory effects.

## Background

Neuroblastoma is the most common solid tumour of childhood. It is a tumour of the postganglionic sympathetic nervous system. The most common genetic features of this tumour are amplification of the oncogene *MYCN*, deletions of part of chromosome arm 1p, gain of parts of 17q and triploidy. *MYCN *amplification, 1p-loss and 17q gain are strongly associated with aggressive tumour and unfavourable outcome for the patient, whereas triploidy is associated with low stage neuroblastomas and a favourable outcome. For review see [1].

A smallest region of overlap of deletion has earlier been determined in primary neuroblastomas by our group to an approximately 2 Mbp region between polymorphic markers D1S214 and D1S244 [2,3]. Although other research groups have defined regions telomeric to marker D1S214 [4], still the major part of all 1p-deleted neuroblastoma tumours does include this region. We hypothesise that this region harbours one or more tumour suppressor genes, which plays an important role in neuroblastoma tumourigenesis. In previous studies a number of different candidate genes from the critical region have been screened for mutations and expression differences. Some examples are *TP73*[5,6], *CORT *[7], *DFFA *[8,9], *UBE4B *[10], *APITD1 *[11], *KIF1B-beta *[12], *KIF1B-alpha *[13], *SDHB *[14], *CDC2L1 *[15,16], *HKR3 *[17], *DAN *[18], *ID3 *[19], *TNFR2 *[20]. However, there is still no conclusive evidence that anyone of these candidate genes could function as a neuroblastoma tumour suppressor gene.

Functional evidence for a neuroblastoma tumour suppressor gene on chromosome 1 was obtained in cell-hybrid studies [21]. The addition of an entire chromosome 1 to a 1p-deleted cell line was shown to induce cell death and/or differentiation indicating that it should be possible to functionally identify the neuroblastoma tumour suppressor gene by introducing candidate genes into 1p-deleted neuroblastoma cells, and then monitoring the cells for reduced cell growth, apoptosis and differentiation.

Non-neural enolase or α-enolase (ENO1) is an enzyme in the glycolytic pathway catalysing the formation of phosphoenolpyruvate from 2-phosphoglycerate. This gene also encodes an alternatively translated product, by initiating the translation at the Met-97 residue in exon 5 [22]. This product was earlier known as the Myc-binding protein-1 (*MBP-1*) [23]. The long 48 kDa form of α-enolase has enzymatic activity and, depending on cell type, a cytoplasmic and/or nuclear localization [24], whereas the shorter 37 kDa form (*MBP-1*) is preferentially localized in the cell nuclei [25]. *MBP-1 *appears to function as a negative regulator of c-myc expression [26]. The c-myc protooncogene is a DNA-binding phosphoprotein that plays an important role in cell growth regulation and differentiation [27]. A major genetic feature of aggressive neuroblastoma tumours is amplification of the oncogene *MYCN*, and this is often associated with loss of chromosome region 1p. Even though there are similarities between the c-myc and *MYCN *oncogenes, there is no evidence that *MBP-1 *is able to interact with *MYCN*. Still it would be of interest to investigate this further by comparing the actions of *ENO1/MBP-1 *in *MYCN*-amplified cells relative to cells with no *MYCN*-amplification.

Enforced overexpression of *MBP-1 *by transfection of *MBP-1 *cDNA into human breast carcinoma cells was shown to inhibit tumour formation in nude mice [28], and the carboxyl-terminal repressor domain of *MBP-1 *has been shown to regress prostate tumour growth in nude mice [29]. Another study has shown that α-enolase is frequently down-regulated in non-small cell lung cancer and that low levels of α-enolase predicts aggressive biological behaviour [30]. Our first hypothesis in designing this study was that the shorter form (*MBP-1*) would play an important role in cell growth regulation in neuroblastoma, considering its localization to the nucleus and its known function as a negative regulator of c-myc expression, and that the longer form (*ENO1*) would not be directly involved in altered cell growth. This hypothesis was proven to be false, since we in this study could show that the longer form (*ENO1*) alone has equal or stronger effect on the cell viability and apoptosis, as do *MBP-1*.

In this paper we have functionally screened six candidate genes from the 1p36.2 region (*CORT*, *DFFA*, *ENO1*, *ICAT*, *PEX14 *and *PGD*) in neuroblastoma cells. We have furthermore performed a more detailed study of *ENO1*, including its shorter translation form *MBP-1*, and a mutated form of *ENO1 *that eliminates the possibility of production of *MBP-1*, by using *in vitro *transcribed mRNA. Our studies demonstrate that *ENO1 *overexpression in both neuroblastoma and 293 cells significantly reduces cell growth and induces apoptosis, implicating *ENO1 *in tumourigenesis. Similar effects could be seen for both *ENO1 *and *MBP-1 *in both *MYCN*-amplified cells (IMR32) and cells with no *MYCN*-amplification (SK-N-AS).

## Methods

### cDNA-constructs

Full length IMAGE cDNA clones of the genes *DFFA *[GenBank:BC000037], *ENO1 *[GenBank:BC009912], *ICAT *[GenBank:BC014300], *PEX14 *[GenBank:BC006327], *PGD *[GenBank:BC000368] (HGMP Resource Centre, UK) and a clone for *CORT *[Genbank:AF013252] (Incyte Genomics, Palo Alto, CA) were used as templates to make a full length PCR product not including the polyA signal and introducing cloning sites in the ends (primer sequences available upon request). For *DFFA*, *ENO1*, *ICAT *and *CORT *a *NotI *site was introduced at the 3' end and an *Eco*RI site at the 5' end. For clones *PGD *and *PEX14 NotI *was used at both ends. The PCR products were digested and ligated into the vector pIRES-EGFP (Clontech, Palo Alto, CA), which includes the CMV promoter for eukaryotic expression and the *EGFP *gene for fluorescence detection, using standard procedures. The resulting clones of *DFFA*, *PEX14 *and *PGD *were also confirmed by restriction digestion (data not shown). Large-scale plasmid DNA preparations were performed using the Qiagen Plasmid Maxi protocol (Qiagen, Hilden, Germany). All constructs were finally sequence confirmed by BigDyeTerminator sequencing, performed on ABI 3700 (Applied Biosystems).

**Figure 1:**
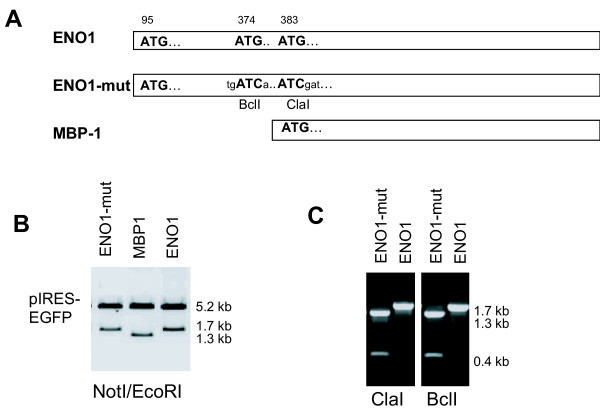
**A. ***ENO1, ENO1*-mut and *MBP-1*-constructs with translation starts (ATG) and mutated (ATG→ATC) sites in *ENO1*-mut at positions 374 and 383 indicated. The mutations introduce *Bcl *I and *Cla *I restriction enzyme sites respectively. **B. **Size determination of cDNA-constructs in pIRES-EGFP-vector by *Not*I/*EcoR*I-digest. **C. **Confirmation of mutations in *ENO1*-mut DNA by *Cla*I and *Bcl*I digests.

A mutated variant of the *ENO1 *cDNA was constructed by introducing two mutations in the *ENO1 *sequence, eliminating the alternative translation start codons of *MBP-1 *(Fig. 1A). The mutations were introduced into the *ENO1 *sequence by PCR using the double-mutated forward primer fp1: 5'-CTGATCATCGAGATCGA TGGAAC-3', in combination with the reverse primer rp2: 5'-ACAAAGAATTCCGG GGATCTAGCCT GTGG-3' for 30 cycles, using the *ENO1*-pIRES-EGFP construct as a template. 0.1 μl of the PCR-product was then used as template in an additional PCR reaction using the primers fp3: 5'-CAGCAGCGGCCGCTTCCTCTC CTAGGCG-3' and rp2: 5'-ACAAAGAATTCCGGGGATCTAGCCTGTGG-3' for 30 cycles of PCR, to incorporate the mutations in the full-length gene product. The *ENO1 *mutant fragment was cloned into the pIRES-EGFP vector by *Eco*RI/*Not*I digestion and religation (Fig. 1B). The mutations were also confirmed by digestion of the PCR product with *BclI *and *ClaI *that only digest the mutated sequence (Fig. 1C).

### Production of *in vitro *transcribed mRNA

A DNA fragment containing the coding region of the *ENO1 *gene linked to the Sp6 promoter sequence and a polyA tail at the end of the fragment was constructed by PCR. The primers used for the *ENO1 *and *ENO1*mut mRNAs were fp: 5'-ATTTAGGTGACACTATAGAAGAGACCCAGTGGCTAGAAGTTCACCATG-3' and rp: 5'-GG(T)_30_CCGGGGATCTAGCCTGTGG-3'. Similarly, the primers for *MBP-1 *mRNA were fp: 5'-ATTTAGGTGACACTATAGAAGAGAACAAGAGAAGATTGACAAACTGATG-3' and rp: 5'-GG(T)_30_CCGGGGATCTAGCCTGTGG-3' using *ENO1*-mut-pIRES-EGFP-vector as a template. The PCR was performed using High Fidelity Taq Polymerase (Roche, Mannheim, Germany) according to supplier's protocol with an annealing temperature of 58°C and 25 cycles of PCR.

500 ng of the PCR-product (663 bp) was used as a template for *in vitro *mRNA using mMESSAGE mMachine (Ambion, Austin, TX). The procedure was according to supplier's protocol using 4 hours incubation at 37°C. The mRNA was DNaseI treated for 15 min at 37°C and LiCl-precipitated before size and concentration evaluation by agarose gel electrophoresis. The control *EGFP *mRNA was produced using an EcoRI-digested Sp6-EGFP vector as template in the *in vitro *mRNA reaction.

The concentrations of *EGFP *and *ENO1 *mRNA preparations were evaluated on agarose and adjusted to be equal for transfection studies.

### Tissue culture

The 1p-deleted neuroblastoma cell lines SK-N-AS and IMR32 (*MYCN*-amplified) (ATCC, Manassas, VA), the human kidney derived cell line 293 and the human erythroleukaemic cell line K562 were cultured in Dulbecco's Modified Eagle Medium (DMEM) with 10% Fetal Calf Serum (FCS) at 37°C using standard procedures. On the day before transfection the cell lines were split into new culture flasks and their viability tested by Trypan Blue staining. Flasks with 60–80% cell confluence were used in transfection experiments. Immediately before the transfection procedure the SK-N-AS cells, the IMR32 cells and the 293 cells were trypsinized and washed twice with PBS and the cell numbers were calculated using Trypan Blue staining. The suspension cells K562 were used only if the viability was 90% or more on the day of transfection.

### Transfection of cDNA vectors

Transfection of the cell lines SK-N-AS and 293 with the cDNA constructs was performed by electroporation using 10 μg of DNA in 4 × 10^6 ^cells in a 0.4 cm cuvette in 500 μl Opti-MEM I (Invitrogen, Carlsbad, CA) at 300 V, 950 μF, ∞ Ω, using a Bio-Rad Gene Pulser II (Bio-Rad, Hercules, CA). The transfection efficiency of SK-N-AS and 293 was tested by transfection of the plasmid pEGFP-N22, followed by estimation of the number of green cells detected by FACS analysis after two days of growth in DMEM 10 % FCS. The transfection efficiency for the SK-N-AS and 293 cells was approximately 90% under these conditions.

For IMR32 cells, transfections were performed using Lipofectamine™ 2000 (Invitrogen). 12 μg of DNA and 25 μl of Lipofectamine™ 2000 in 1200 μl Opti-MEM I were used for transfecting 10^6 ^cells in 5 ml of DMEM, 10% FCS. Transfection efficiency was estimated to be 60 % by fluorescence microscopy of pEGFP-N22 transfected cells, two days after transfection.

Transfection with all plasmid constructs was performed twice using SK-N-AS cells. The *ENO1 *construct and empty vector control were also transfected in four different experiments into SK-N-AS cells and twice into IMR32 and 293 cells.

### Transfection of mRNA

The transfections were performed in a 12-well plate using 4 × 10^5^cells/well for SK-N-AS and K562 cells and 7 × 10^5 ^cells/well for 293 cells, all in 2 ml DMEM with 10% FCS per well. For each transfection a total of 1.5 μg of mRNA (A: 1.5 μg *EGFP *mRNA; B: 0.3 μg *ENO1 *mRNA + 1.2 μg *EGFP *mRNA; C: 1.5 μg *ENO1 *mRNA) and 5 μl Lipofectamine™ 2000 were used. The mRNA and the Lipofectamine™ 2000 were incubated together for 30 min in 200 μl Opti-MEM I and then added to the cell suspensions. The cells were immediately divided into 96-well plates at 10^4 ^cells/well (SK-N-AS, K562) or 1.7 × 10^4 ^cells/well (293) (50 μl/well), and incubated at 37°C for 5 hours before an extra 50 μl DMEM with 10% FCS was added. The cells were incubated for 1–4 days in 37°C for cell growth studies.

### Apoptosis assay

After transfection, the cells were allowed to attach for two hours in a tissue culture plate (6 well) before the medium was replaced by fresh DMEM 10% FCS. Then the cells were grown for two days before all cells, both in medium and attached cells (trypsinized) were washed with PBS and fixed in a final concentration of 2% paraformaldehyde.

The fixed cells were TUNEL stained using the In Situ Cell Death Detection Kit TMR-red kit (Roche, Mannheim, Germany) according to supplier's protocol. This stains all apoptotic cells red. The cells were then analysed by FACS analysis.

### Cell growth assay

After transfection, the cells were divided into 96-well plates. Cells with each different construct were divided into 8 wells/plate, in four different plates with 10^4 ^cells in 100 μl DMEM 10 % FCS per well. One plate was used every day for four days after transfection to monitor cell growth. 20 μl of CellTiter96 Aqueous One Solution (Cell Proliferation Assay, Promega, Madison, WI) was added to each well and incubated in a humid dark chamber at 37°C for four hours. The absorbance was detected at 490 nm by an ELISA-reader (Labsystems Multiscan RC, Helsinki, Finland). The results were compared to a standard curve to calculate the number of viable cells per well each day. The mean number of cells in the 8 wells evaluated per day per construct was used in evaluating the results.

The transfection efficiency was evaluated one day after transfection using fluorescence microscopy, by counting the number of green cells in the pEGFP-N22 or *EGFP *mRNA experiments.

### Patient material

Frozen (-70°C) tumour samples from 44 neuroblastoma patients; 4 Stage 1 (1 1p-deleted), 4 Stage 2, 14 Stage 3 (7 1p-deleted), 20 Stage 4 (14 1p-deleted), 2 Stage 4S; 2 ganglioneuromas and 2 teratomas (1 1p-deleted) were used in the study.

The tumours were staged according to the International Neuroblastoma Staging System criteria [31,32]. The 1p-deletion status has been previously evaluated [2,16]. DNA was extracted from patient samples using standard procedures.

### Expression studies of *ENO1*

#### cDNA preparation

Total RNA was extracted from frozen neuroblastoma tumour tissue from 11 primary neuroblastomas; 4 Stage 2 tumours from patients with no evidence of disease after treatment; 7 Stage 4 tumours (4 1p-deleted), all from patients dead of disease and 7 neuroblastoma cell lines (4 1p-deleted) using RNeasy RNA extraction kit (Qiagen). 2.4 μg total-RNA of each sample was reversed transcribed to cDNA using Superscript II (Amersham, Buckinghamshire, UK) and random hexamer primers, all according to supplier's protocol.

#### Real time RT-PCR

TaqMan primers and probes were derived from the commercially available TaqMan^® ^Assays-on-Demand Gene Expression Products. To select the most appropriate endogenous control for the real-time RT-PCR quantification analysis of neuroblastoma primary tumours, we used TaqMan Human Endogenous Control Plate (catalog number: 4309921, Applied Biosystems, Foster City, CA). Analysis was performed according to supplier's protocol.

To select the most appropriate endogenous control for the real-time PCR quantification analysis, we tested eight different primary NB samples of different stages for their expression levels of ten commonly used housekeeping genes. *GUSB *(β-glucuronidase) and *B2M *(β_2_-microglobulin) showed least variations in ΔC_T _levels, and were expressed at constant levels in all samples regardless of NB-stage. *GUSB *was selected, and further used as an internal reference for normalization in the real-time PCR quantification analysis [9].

Real-time RT-PCR was performed in 384-well plates using ABI PRISM^® ^7900HT Sequence Detection System (Applied Biosystems). Amplification reactions (10 μl) were carried out in duplicate with 1 μl of 1:10 diluted template cDNA according to manufacturer's protocol (Applied Biosystems). In each assay, a standard curve with six cDNA dilutions was recorded and two non-template controls were included.

Quantification was performed by the standard-curve method. The mean C_T_-value for duplicates was calculated, and the gene concentration (or gene copy number) of test samples was interpolated based on standard curves. All samples were normalized by dividing the concentration of the test gene with the concentration of the housekeeping gene β-glucuronidase (*GUSB*) in the same cDNA sample.

#### Statistical analysis

Statistical analysis was done with Student's two-sided t-test on each group of tumors; low-stage and high stage tumors; and for 1p-deleted and not 1p-deleted tumors for the expression study.

In the transfection study, Student's two-sided t-test was used to compare cell numbers in each experiment at 1, 2, 3 and 4 days after transfection.

#### Mutation screening

Mutation screening of all exonic sequences, selected intronic sequences and the promoter region of *ENO1 *was performed on genomic DNA from 48 primary neuroblastoma tumours of all different stages. The different regions were amplified by PCR and the PCR products were screened for mutations using either BigDyeTerminator sequencing, performed on ABI 3700 (Applied Biosystems) and/or DHPLC (WAVE) (Becton, Dickinson and Company) all according to general protocols of the suppliers. PCR-primers were designed to include the entire exon plus exon-intron boundaries. PCR primers and amplification data are available on request.

## Results

### Functional studies

**Figure 2:**
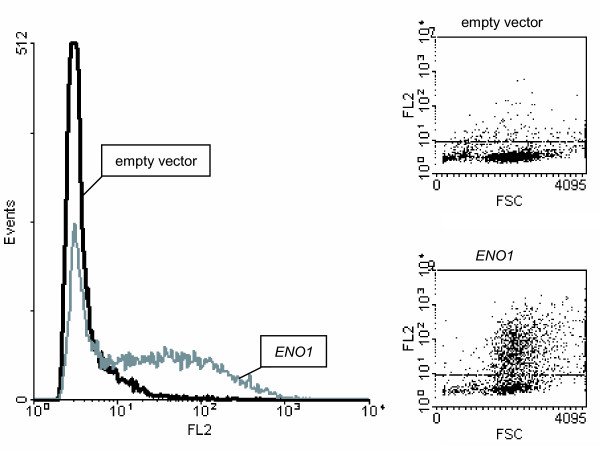
FACS results of SK-N-AS cells two days after transfection of empty pIRES-EGFP-vector (black line) and *ENO1*-pIRES-EGFP (grey line). The cells were TUNEL stained using TMR-red labelled nucleotides to label all apoptotic cells red. There is a clear increase of the number of red cells (FL2) in *ENO1 *transfected cells compared to empty vector control.

TUNEL staining, followed by FACS-analysis, of *ENO1*-transfected SK-N-AS neuroblastoma cells two days after transfection showed a higher degree of apoptotic cells (59%) than with the empty vector control (9%) or any of the other constructs tested (8–26% apoptotic cells), (Table 1, Fig. 2).

**Figure 3:**
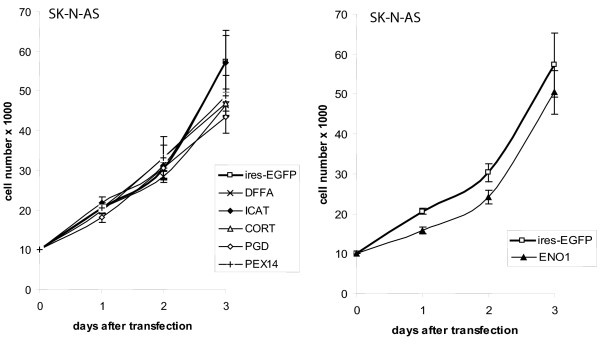
Cell growth curves of cDNA-transfected cells monitored for 3 days after transfection. The average number of cells in 8 separate experiments is shown at each time point for each construct. Standard deviation is indicated with a bar. **A: **Cell growth after transfection of five different constructs compared to empty vector (pIRES-EGFP) control in SK-N-AS cells. Using Students T-test, no significant differences in cell number compared to control cells could be detected for the first two days after transfection for any of the constructs, three days after transfection DFFA (p = 0.002), CORT (p = 0.004) and PGD (p < 0.001) showed significant difference. **B: **Cell growth after transfection of ENO1 compared to empty vector control. Significant differences in cell number was detected day 1 (p < 10^-8^) and day 2 (p < 10^-4^), the difference was not significant day 3 after transfection.

The DNA transfection studies also showed that the *ENO1 *gene did have the strongest and most consistent effect (approximately 20% reduction) on the growth rate of 1p-deleted neuroblastoma cells (SK-N-AS and IMR32) compared to an empty vector control (Table 2, Fig. 3). In the same assay, *DFFA*, *PGD *and *PEX14 *showed an approximately 10% reduction in cell growth (SK-N-AS), *ICAT *did not show any reduction and *CORT *showed very variable effects (from -10% to 18% reduction) (Table 2, Fig. 3). Transfection experiments using the *ENO1*-gene compared to empty vector control were also performed in the 1p-deleted and *MYCN*-amplified neuroblastoma cell line IMR32 and the non-neuroblastoma cell line 293. These experiments showed that *ENO1 *reduced cell growth by 20–30 % in all three cell lines (Table 2).

Interpretation of cell growth studies after transfection of plasmids carrying various genes may be complicated by the fact that only a fraction of cells express the relevant gene, whereas the growth of the remaining cells may continue unaffected. In an effort to ensure that most cells express the relevant gene after transfection, we have also investigated transfection of each one of the genes as *in vitro *transcribed mRNA. Preliminary studies with *EGFP*-mRNA showed that the transfection efficiency, as determined by fluorescence microscopy 24 hours after transfection, was 90% for SK-N-AS cells, 60% for K562 cells and 90% for 293 cells.

**Figure 4:**
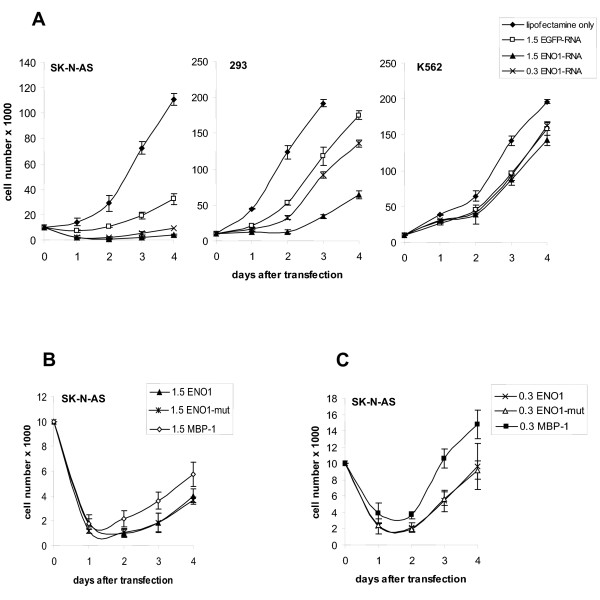
Cell growth curves of cell lines SK-N-AS, 293 and K562 after transfection of *in vitro *transcribed mRNA and monitoring for 4 days after transfection. The average number of cells in 8 separate experiments is shown at each time point for each construct. Standard deviation is indicated with a bar. **A: **Two different amounts of mRNA have been used for *ENO1*-mRNA: 1.5 (1.5 μg *ENO1*-mRNA) or 0.3 (0.3 μg *ENO1*-mRNA + 1.2 μg *EGFP*-mRNA), compared to 1.5 μg *EGFP*-mRNA and lipofectamine only. There is strong statistical significance (p << 0.001) for difference in cell numbers between the *ENO1*-mRNA-experiments and the *EGFP*-mRNA experiments in SK-N-AS and 293-cells (exact p-values for each day are listed in Table 3), in K562 cells the difference was not significant. **B: **Cell growth curves of SK-N-AS cells after transfection of 1.5 μg of *ENO1*, *ENO1-mut *and *MBP-1 *mRNAs and monitoring for 4 days. The difference in cell numbers between *ENO1 *and *MBP-1 *was significant for day 2–4 (p = 0.02), *ENO1 *and *ENO1-mut *did not significantly differ. **C: **Cell growth curves of SK-N-AS cells after transfection of 0.3 μg *ENO1*, *ENO1-mut *and *MBP-1 *mRNAs and monitoring for 4 days. The difference in cell numbers between *ENO1 *and *MBP-1 *was significant for day 2 (p = 0.006), 3 (p = 0.001) and 4 (p = 0.02). *ENO1 *and *ENO1-mut *did not significantly differ.

Our mRNA-transfection experiments showed that by adding 1.5 μg of *ENO1*- or *ENO1*-mut-mRNA to the neuroblastoma cell line SK-N-AS, the cell numbers were reduced by 60% after 24 hours, and by 80% after 2–4 days of incubation, compared to *EGFP*-mRNA transfected cells (Table 3, Fig. 4A &4B). On the other hand, 1.5 μg *MBP-1*-mRNA reduced the cell growth rate by 50% after 24 hours and 70% 2–4 days after transfection (Table 3, Fig. 4B), indicating that *MBP-1*-mRNA is less effective than the full length transcripts.

When 0.3 μg *ENO1*-mRNA or *ENO1*-mut-mRNA mixed with 1.2 μg of *EGFP*-mRNA were used, the reduction in cell numbers after 24 hours was 40%, and 60% respectively after 2–4 days. As with the 1.5 μg experiment, 0.3 μg *MBP-1*-mRNA mixed with 1.2 μg of *EGFP*-mRNA gave a lower reduction of 30% after 24 hours, and 40% 2–4 days after transfection (Table 3, Fig. 4C).

In 293 cells a large reduction in cell numbers in the 1.5 μg *ENO1*-mRNA experiments could be seen after 24 hours (50%), and after 2–4 days (90%) compared to *EGFP *transfected control cells (Table 3, Fig. 4A). For 1.5 μg *MBP1*- or *ENO1*-mut-mRNA the reduction in 293 cells was 40% after 24 hours, and 70–80% 2–4 days after transfection. A parallel experiment with 0.3 μg *ENO1*-mRNA showed about 60% reduction 2–4 days after transfection compared to control cells, while in the same experiment *ENO1*-mut and *MBP-1 *mRNAs showed a 30% and a 50% reduction respectively (Table 3).

In K562 cells, the cell numbers 24 hours after transfection were not significantly different between experiments (Table 3).

### Expression studies

**Figure 5:**
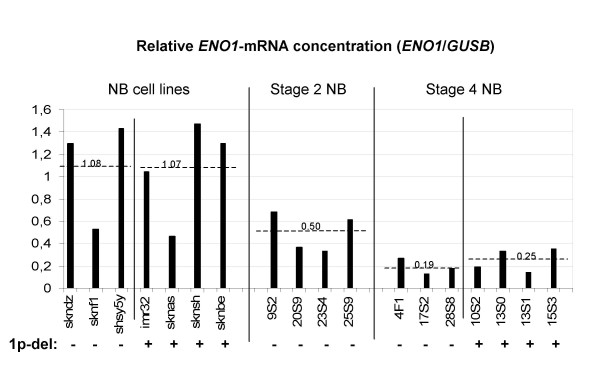
Expression of *ENO1 *in neuroblastoma cell lines and primary neuroblastoma tumours using real-time-PCR. Relative concentrations of *ENO1*-mRNA in relation to the concentration of the housekeeping gene *GUSB *for each sample are indicated on the y-axis. The samples are grouped into five groups: neuroblastoma cell lines, with and without 1p-deletion; stage 2 tumours; stage 4 tumours, with and without 1p-deletion. The 1p-deletion-status of each sample is indicated at the bottom. For each group the average value is indicated by a dotted line. A difference in expression could be detected between stage 2 tumours and stage 4 tumours (p = 0.01). No difference in expression could be detected between 1p-deleted samples and not 1p-deleted samples.

No difference in expression levels could be detected between 1p-deleted cell lines (average relative concentration of 1.07) and 1p-intact cell lines (average relative concentration of 1.08). Also in Stage 4 neuroblastoma primary tumours, no difference in *ENO1*-expression could be detected between 1p-deleted (average relative concentration 0.25) and 1p-intact samples (average relative concentration of 0.19) (Fig. 5).

Real time-PCR studies of the *ENO1 *gene on cDNA-samples showed a 55% reduction (p = 0.01) of *ENO1*-mRNA levels in stage 4 neuroblastoma tumours compared to stage 2 tumour levels (average relative concentration of 0.5 in stage 2 tumours compared to 0.23 in stage 4 tumours). This reduction was independent on the 1p-deletion status of the tumour sample (Fig. 5).

### Mutation screening

The mutation screening of the promoter region and of all exonic sequences of *ENO1*, including exon/intron boundaries, and of introns 5–7 in 48 primary neuroblastoma samples of different stages did not reveal any mutations (data not shown).

## Discussion

The aim of this study was to functionally identify a tumour suppressor gene in the commonly deleted region 1p36 in advanced stage neuroblastoma tumours. We were in fact able to identify one gene, *ENO1*, located in the smallest region of overlap of deletion in neuroblastoma, which did seem to have tumour suppressor activity. That is, by introducing this gene as a cDNA construct into neuroblastoma cells, the number of viable cells over time was reduced, and the number of apoptotic cells was increased. These two results are probably just two ways of monitoring the same thing. A higher degree of cell death will obviously slow down number of viable cells over time. We also tested the ability of *ENO1 *cDNA to slow down cell growth in other cell types that did not have a deletion of 1p. Here we choose the 293 cell line (human transformed kidney cells). Other cells that could add valuable information would be a neuroblastoma cell line with intact 1p (such as SK-N-SH or SK-N-F1), we were though unable to get a satisfactory transfection efficiency in these cell lines for this study, and thus choose to use the 293 cells that worked very well, that also adds valuable information about the general effect of *ENO1 *on cells with intact 1p. In this study we monitor viable cells over time after transfection, thus it is crucial to have high percentage of transfected cells otherwise your experiment would easily be overgrown by the untransfected cell population. After transfection of *ENO1 *into the 293 cells the same results were obtained as with the neuroblastoma cell lines, that is, the number of viable cells over time was reduced by 20–40 %. These results are concordant with the results of Ray and co-workers that transfection of *MBP-1 *into human breast carcinoma cells showed reduction of cell growth and inhibition of tumour formation in nude mice [28]. *MBP-1 *was later demonstrated to be a shorter translational variant of the *ENO1 *gene [22,25]. The localization of the gene in the 1p36.2 chromosomal region makes it a very interesting gene for neuroblastoma tumourigenesis and one hypothesis is that one deleted copy of *ENO1 *in the neuroblastoma cells would result in lower levels of α-enolase, and thus these cells would have a stronger response when more *ENO1*-mRNA is added. However, in this study we did see an equally strong effect in the chromosome 1 intact cells 293. Maybe the level of ENO1 is crucial in the cells, and by adding too much of this gene product shifts the balance easily and makes the cell go into apoptosis. Also we could see in the *ENO1*-expession study that the 1p-deleted cell lines did not have lower levels of *ENO1*-mRNA compared to the 1p-intact cell lines. This adds to the hypothesis that the level of *ENO1 *expression might be crucial. One hypothesis was that it might be a difference in the response to added *ENO1 *in *MYCN*-amplified cells (IMR32) and cells with normal *MYCN *(SK-N-AS), since the shorter form of *ENO1 *(*MBP-1*) appears to function as a negative regulator of c-myc expression [26]. No difference could however be seen in the response in these two cell lines, indicating that *ENO1 *has growth inhibitory properties independent of effects on *MYCN *regulation.

We could not detect a consistent reduction in cell growth with the other gene constructs tested. Also we could not detect any clear increase in apoptotic cell numbers (Table 1), and no obvious signs of differentiation, although our data do not fully exclude a role for one or more of these genes in the development of neuroblastoma. Noteworthy is also another gene, *APITD1*, recently localised to this region of interest. *APITD1 *could be shown to slow down cell growth and induce apoptosis in neuroblastoma cells [11]. We can hypothesise that if a number of genes in this specific region have tumour suppressor activity, a small reduction in expression of all these genes can give a substantial effect on cell growth. A recent study has indeed shown that there are numerous genes from the 1p36-region down-regulated in aggressive neuroblastoma tumours [33].

In order to further investigate the ability of *ENO1 *to slow down cell growth, and to compare the different translation products of *ENO1 *in a functional manner, we constructed one mutated form of *ENO1*, *ENO1*-mut, which would eliminate the possibility for the alternative form *MBP-1 *to be translated (Fig. 1). We used the *ENO1 *cDNA vector as a template for in vitro mRNA to make *ENO1 *mRNA and a shorter form only including the *MBP-1*-part of the gene to make *MBP-1*-mRNA. The *ENO1*-mut construct was used as a template for in vitro *ENO1*-mut-mRNA.

We could show that *ENO1 *mRNA has a strong, dose dependent, inhibitory effect on cell growth in neuroblastoma cells (SK-N-AS). By adding equal amounts of *ENO1*-mRNA and control-mRNA (*EGFP*) to the cells, the number of viable cells in the cell culture was decreased by 80% two days after transfection in the *ENO1*-mRNA-experiment. If 4/5 of the *ENO1*-mRNA was exchanged with control-mRNA the decrease was 60% two days after transfection (Table 3, Fig. 4). This indicates that *ENO1 *acts in a dose dependent manner, suggesting that cellular levels of ENO1 might play a crucial role in regulating apoptosis and proliferation in neuroblastoma cells.

Surprisingly the mutated *ENO1*-mut-mRNA showed very similar effects as the wild type *ENO1*-mRNA. This might be explained by the fact that *ENO1 *in fact can bind to the same sites as *MBP-1 *and act as a transcription factor, even though not as efficiently [22]. Maybe by adding very large amounts of mRNA, this difference is overruled. However, an alternative explanation is that the enolase itself has tumour suppressor activity. This is supported by the observation that 0.3 μg *ENO1 *mRNA induces a stronger inhibition in cell growth rate (60%) than the same amount of *MBP-1*-mRNA (40%) in neuroblastoma cells.

A strong effect could also be seen in 293-cells by adding *ENO1*-mRNA. 80–90% reduction was observed in the 1.5 μg mRNA experiment, while a lower reduction (60%) was observed when 0.3 μg mRNA was used, as was the case with SK-N-AS cells.

A difference between the different mRNAs could also be seen in the 1.5 μg experiment where *MBP-1 *and *ENO1*-mut showed a weaker effect than *ENO1*. Also in the *ENO1*-mut and *MBP-1 *experiments, a smaller effect was observed with a lower amount of mRNA (0.3 μg). We conclude that over-expression of *ENO1 *has the same effect in both neuroblastoma cells and transformed kidney cells, and that *ENO1 *can function as a general tumour suppressor.

However, the transfection of *ENO1*-mRNA into K562 cells, a human erythroleukemia cell line, shows only minor differences with control mRNA (Table 3, Fig. 4). This indicates that *ENO1*-mRNA has no general toxicity to cells but seems to work through one or more specific pathways. *ENO1*-mRNA does not seem to activate any cell death or growth suppression pathway in the lymphoblast cell line K562, as it seems to do in the neuroblastoma cell line SK-N-AS and the transformed kidney cell line 293. But these are indeed very different types of cells, and are likely to be defective in different cellular pathways.

In order to see whether *ENO1 *was equally expressed in both low stage and advanced neuroblastomas, and also to compare this with the mRNA-levels in neuroblastoma cell lines, we conducted a real-time-PCR-study using Taqman. Surprisingly no difference in the *ENO1*-mRNA levels could be detected between 1p-deleted tumours and 1p-intact tumours, neither in the cell lines nor in primary Stage 4 neuroblastoma tumours (Fig. 5). The loss of one allele seems either to be compensated in these cells, or the levels of *ENO1*-mRNA in the aggressive Stage 4 tumours are suppressed by a different mechanism in the 1p-intact Stage 4 tumours. Even though small sample number (11 primary tumours), we could detect a difference (p = 0.01) in the levels of *ENO1*-mRNA in Stage 4 neuroblastomas compared to Stage 2 neuroblastomas (Fig. 5). This still needs further studies to be confirmed but one could speculate that the *ENO1*-mRNA level might be of importance in the aggressiveness of neuroblastoma tumours, as seen in non-small cell lung cancer [30]. The cell lines showed a higher relative concentration of *ENO1 *than any of the primary tumours (Fig. 5). This might be explained by the different surroundings and state of cells in culture compared to primary tumour cells (Fig. 5). The fact that by adding extra *ENO1*-mRNA to the cell lines, even though they expressed *ENO1 *already, made them apoptotic, adds to the hypothesis that the levels of this mRNA might be crucial.

We also did a mutation screening of the exonic sequences of *ENO1*, including exon/intron boundaries, and of the intronic sequences between exons 4–8, in order to cover the region around the alternative translation start of *MBP-1*, in genomic DNA of 44 primary neuroblastoma tumours of all different stages. However, no mutations could be found in these regions, indicating that changes in the level of *ENO1 *expression may result from other types of mutations or from changes in regulatory mechanisms.

## Conclusion

In conclusion, our studies demonstrate that *ENO1*, a gene mapping to a region commonly deleted in advanced neuroblastoma tumours, can act as a strong tumour suppressor by slowing down the cell growth and inducing apoptosis. Although we could not identify any mutations in the coding sequence and immediate promoter region of the gene in 48 primary tumour samples, our studies suggest that variations in the level of expression of the gene can play a role in regulating apoptosis and cell proliferation in neuroblastoma. To prove the hypothesis of the level of *ENO1 *expression being crucial, future investigations could involve silencing of *ENO1 *in neuroblastoma cells *in vitro*, by gene knock down experiments and/or siRNA targeted to the *ENO1*-mRNA, to detect whether this, opposite adding extra *ENO1*-mRNA, increases the growth rate. Deletion of the 1p chromosomal region is obviously one way to achieve a reduction in expression of *ENO1*, but it is not clear whether a reduction in the expression of the second allele is also necessary through other mechanisms in neuroblastoma.

## Competing interests

The author(s) declare that they have no competing interests.

## Authors' contributions

KE carried out the functional studies, the expression studies, the exon sequencing, study design and drafted the manuscript. CK and HC carried out the promoter- and intron sequencing. FZ participated in the in vitro mRNA-design and carried out FACS-analysis. LL participated in tissue culture and FACS analysis. TM participated in the design of the mutation analysis study. PI participated in the design and coordination of the functional study and helped to draft the manuscript.

All authors read and approved the final manuscript.

## Pre-publication history

The pre-publication history for this paper can be accessed here:



## Figures and Tables

**Table 1:** Induction of apoptosis two days after transfection of cDNA constructs in SK-N-AS neuroblastoma cells.

**Gene**	**% Apoptotic cells**
Empty vector	9 %
*ENO1*	59 %
*CORT*	19 %
*DFFA*	21 %
*ICAT*	26 %
*PEX14*	8 %
*PGD*	9 %

**Table 2:** Effects of various cDNA constructs on cell growth after transfection into neuroblastoma and other tumour cell lines.

**Cell Line**	**Gene**	**day**	**% decrease in number of cells +/-SD**	**number of experiments**	**p-value: test**
SK-N-AS	Empty vector (control)		-	3 × 8	
	*ENO1*	1	22 +/- 8	3 × 8	**< 0.001**
		2	21 +/- 4	3 × 8	**0.004**
		3	13 +/- 3	3 × 8	**0.04**
		4	27 +/- 12	3 × 8	**< 10^-5^**
	*CORT*	1	3 +/- 7	1 × 8	> 0.05
		2	-10 +/- 14	1 × 8	> 0.05
		3	18 +/- 14	1 × 8	**0.004**
		4	4 +/- 8	1 × 8	> 0.05
	*DFFA*	1	2 +/- 8	1 × 8	> 0.05
		2	6 +/- 11	1 × 8	> 0.05
		3	19 +/- 12	1 × 8	**0.002**
		4	14 +/- 6	1 × 8	**< 10^-6^**
	*ICAT*	1	-6 +/- 10	1 × 8	**0.04**
		2	-3 +/- 9	1 × 8	> 0.05
		3	0 +/- 23	1 × 8	> 0.05
		4	-2 +/- 6	1 × 8	> 0.05
	*PEX14*	1	1 +/- 10	1 × 8	> 0.05
		2	-9 +/- 18	1 × 8	> 0.05
		3	15 +/- 18	1 × 8	**0.02**
		4	9 +/- 7	1 × 8	**0.001**
	*PGD*	1	12 +/- 8	1 × 8	**< 0.001**
		2	-1 +/- 16	1 × 8	> 0.05
		3	24 +/- 15	1 × 8	**< 0.001**
		4	11 +/- 7	1 × 8	**< 10^-4^**
IMR32	*ENO1*	1	12 +/- 16	1 × 8	**0.02**
		2	23 +/- 14	1 × 8	**0.01**
		3	14 +/- 14	1 × 8	**< 0.001**
		4	18 +/- 8	1 × 8	**< 10^-5^**
293	*ENO1*	1	15 +/- 20	1 × 8	**0.03**
		2	49 +/- 29	1 × 8	**0.006**
		3	19 +/- 10	1 × 8	**< 0.001**
		4	46 +/- 9	1 × 8	**< 10^-7^**

**Table 3:** Effects of *in vitro *transcribed ENO1 mRNA constructs on cell growth after transfection into neuroblastoma (SK-N-AS), 293 and K562 cell lines.

**cell line**		**SK-N-AS**			**293**			**K562**		
**mRNA**	**day**	**% decrease in ** **number of** **cells +/-SD**	**no of exp**	**p-value test**	**% decrease in** **number of** **cells +/-SD**	**no of exp**	**p-value test**	**% decrease in ** **number of** **cells +/-SD**	**no of exp**	**p-value test**

1.5 μg ***EGFP***		-	4 × 8		-	3 × 8		-	2 × 4	
1.5 μg ***ENO1***	1	58 +/- 12	4 × 8	**< 10^-5^**	52 +/- 11	3 × 8	**< 10^-3^**	-11 +/- 19	2 × 4	> 0.05
	2	81 +/- 11		**< 10^-9^**	85 +/- 8		**< 10^-5^**	13 +/- 34		> 0.05
	3	79 +/- 12		**< 10^-9^**	89 +/- 16		**< 10^-5^**	9 +/- 11		> 0.05
	4	76 +/- 17		**< 10^-9^**	87 +/- 20		**< 10^-5^**	10 +/- 9		**0.03**
0.3 μg ***ENO1***	1	39 +/- 25	3 × 8	**0.02**	38 +/- 27	2 × 8	**< 10^-4^**	-14 +/- 14	1 × 4	**0.04**
	2	63 +/- 15		**< 10^-5^**	64 +/- 36		**< 10^-4^**	6 +/- 11		> 0.05
	3	60 +/- 11		**< 10^-5^**	60 +/- 54		**< 10^-4^**	4 +/- 10		> 0.05
	4	64 +/- 13		**< 10^-3^**	59 +/- 52		**< 10^-5^**	-3 +/- 9		> 0.05
1.5 μg ***ENO1-mut***	1	65 +/- 26	2 × 8	**0.007**	44 +/- 9	1 × 8	**< 10^-3^**	-6 +/- 14	2 × 4	> 0.05
	2	84 +/- 8		**< 10^-3^**	77 +/- 4		**< 10^-6^**	20 +/- 8		**0.004**
	3	84 +/- 8		**< 10^-4^**	73 +/- 6		**< 10^-4^**	16 +/- 13		**0.03**
	4	84 +/- 7		**< 10^-3^**	63 +/- 6		**< 10^-6^**	5 +/- 11		> 0.05
0.3 μg ***ENO1-mut***	1	66 +/- 8	1 × 8	**< 10^-3^**	20 +/- 13	1 × 8	**0.02**	-18 +/- 13	1 × 4	**0.01**
	2	82 +/- 4		**< 10^-6^**	50 +/- 7		**< 10^-4^**	8 +/- 12		> 0.05
	3	70 +/- 7		**< 10^-4^**	28 +/- 15		**0.007**	9 +/- 13		> 0.05
	4	72 +/- 6		**< 10^-4^**	29 +/- 6		**< 10^-4^**	-1 +/- 11		> 0.05
1.5 μg ***MBP-1***	1	45 +/- 21	4 × 8	**< 10^-3^**	41 +/- 8	3 × 8	**0.005**	-9 +/- 16	2 × 4	> 0.05
	2	67 +/- 13		**< 10^-7^**	75 +/- 10		**< 10^-4^**	10 +/- 12		> 0.05
	3	68 +/- 15		**< 10^-6^**	82 +/- 25		**< 10^-4^**	1 +/- 8		> 0.05
	4	64 +/- 20		**< 10^-7^**	76 +/- 31		**< 10^-4^**	5 +/- 9		> 0.05
0.3 μg ***MBP-1***	1	25 +/- 18	3 × 8	> 0.05	50 +/- 38	2 × 8	**0.02**	-14 +/- 14	1 × 4	**0.04**
	2	47 +/- 18		**0.001**	56 +/- 18		**< 10^-5^**	4 +/- 18		> 0.05
	3	38 +/- 7		**0.001**	44 +/- 46		**< 10^-3^**	-11 +/- 17		> 0.05
	4	37 +/- 19		**0.006**	42 +/- 34		**< 10^-4^**	-2 +/- 8		> 0.05
